# Bacterial community dynamics with rhizosphere of Calotropis procera and Senna alexandrina desert plants in Saudi Arabia

**DOI:** 10.6026/97320630016567

**Published:** 2020-08-31

**Authors:** Diana AH Al-Quwaie

**Affiliations:** 1 Department of Biological Sciences, Rabigh College of Science and Arts, King Abdulaziz University (KAU), Rabigh, Saudi Arabia

**Keywords:** OTUs, Microbiome, Rhizobacteria, Microbial abundance, Plant growth rate

## Abstract

It is of interest to study the rhizobacteria associated with two different desert wild plants, e.g., Calotropis procera and Senna alexandrina compared with bulk soil sample in order to identify signatures of microbes in rhizospheres of the two plants and detect influence of soil microbiome in drawing soil architecture. Analysis of deep sequencing microbial dataset indicated occurrence of 296,642 sequence tags assigned 5,210 OTUs (operational taxonomic units). Species richness in control sample was higher than those of either plant's rhizosphere, while microbial abundance was lower. Principal coordinate analysis (PCoA) plot indicated complete separation of microbiome diversity among groups. Abundances of Pseudomonas stutzeri and Virgibacillus koreensis increased in the rhizosphere of C. procera compared with that of S. alexandrina, while those of Streptococcus sobrinus, Veillonella parvula and unassigned species of Sphingomonas genus increased in rhizosphere of S. alexandrina. Unassigned species of genera Marinobacter, Porticoccus and Alcanivorax only exist in rhizosphere microbiome of C. procera, while unassigned species of genus Pseudomonas only exists in rhizosphere microbiome of Senna alexandrina. High abundances of the two microbes Pseudomonas stutzeri and Virgibacillus koreensis in rhizosphere of C. procera allow the plant to grow well under both normal and saline condition. Also, Marinobacter, Porticoccus and Alcanivorax genera only exist in rhizosphere microbiome of C. procera. These microbes produce siderophores that protect plant from pathogens. Data shows that C. procera might be more protected from microbial pathogens compared with S. alexandrina. The differential abundances or exclusive presence of soil microbes reflect the ability of plant species to survive under biotic and abiotic stresses. Results imply that rhizospheric microbes can be used as biomarkers of plant growth rate and the ability to survive under harsh conditions.

## Background

Soil harbors highly diverse microbial communities that either grow on their own or interact with surrounding plant roots within an environmental narrow zone called rhizosphere [1,2]. This zone is a hot spot for numerous microorganisms representing the most complex ecosystems on Earth [3,4]. These microorganisms include bacteria, fungi, nematodes, protozoa, algae, viruses, archaea, and arthropods. Beneficial rhizosphere organisms that improve plant growth and health include nitrogen-fixing bacteria and plant growth-promoting rhizobacteria (PGPR). However, other rhizosphere organisms can have negative influence on plant growth and health. In addition, there are microorganisms that can act as pathogens to human [5]. At rhizosphere zone, plants interact with soil microbiomes of which root exudates contribute to bacterial community differentiation [6-8]. This takes place by stimulating/repressing bacterial growth and subsequent alteration of soil microhabitat [3,9, 10]. Soil type and plant genotype as well as plant developmental stage have been defined as the major contributors in shaping such rhizobacterial communities [11-13]. In addition, microbe-microbe interactions (biotic relationships), soil pH, carbon content and mineral constitution (abiotic relationships) also contribute to shaping bacterial diversity and abundance in the rhizosphere [14- 16].

The contribution of rhizobacteria in improving health and growth of many agroecosystems, mainly crop plants, became a major interest for scientists [17- 19]. However, studies of soil bacterial communities associated with native vegetation are scarce [4,20- 22], especially in extreme environments such that of the desert land in Saudi Arabia [15,23, 24]. In extreme environment, tree plants play important role in stabilizing soil architecture and microbes, increasing nutrient availability and water-holding capacity, in addition to avoiding soil erosion [25]. The ability of plants to adapt and survive at inadequate environmental conditions depends on their association with a specific rhizospheric microbiomes [26-28]. The study of rhizobacteria is crucial to understanding their ability to confer tolerance to high levels of abiotic stress such as high salinity, drought, high temperature, UV and low nutrient availability [29]. In Saudi Arabia, soil microbial communities are extremely native mostly because habitat is considered as the driest edge of life [30-32]. Further, little is known about the diversity of bacterial communities associated with plants in such regions [22]. Therefore, it is of interest to document the dynamics in bacterial community associated with rhizosphere of Calotropis procera and Senna alexandrina desert plants in Saudi Arabia.

## Methodology

### Sample collection:

A total of four soil rhizosphere samples associated with two desert plants namely Calotropis procera and Senna alexandrina, two samples each (Figures S2 and S3, respectively), were collected. In addition, one plant-free soil sample was collected away from assigned location where no plants are growing within a circle of five meters. Sampling was carried out during January 2019 from a location in Bahra near Jeddah, Saudi Arabia with latitude: 21 23' 26.94" N and longitude: 39 21' 21.822" E and altitude: 93.93 m above sea level (Figure S1). An amount of 100 g soil was collected 15 cm beneath the first layer of altered soil for the different samples. Samples were immediately kept in dry ice and stored at -80°C until further analysis.

### DNA extraction and deep sequencing of 16S rRNA partial gene:

Genomic DNA was extracted from soil samples using the DNeasy PowerSoil Pro Kit (Qiagen, Germany) following manufacturer's instructions. DNA purity was evaluated via A260/A280 ratio using NanoDrop 7000 spectrophotometer (Thermo Fisher Scientific, Waltham, MA, USA), and DNA integrity was checked by 1% agarose gel electrophoresis. Amplification of the V3-V4 region of bacterial 16S rRNA was performed using the universal primers 338F (5'-ACTCCTACGGGAGGCAGCA-3') and 806R (5'-GGACTACHVGGGTWTCTAAT-3') with a barcode in the forward primer. PCR program was: initial denaturation at 95°C for 5 min; 25 cycles of denaturation at 95°C for 30s, annealing at 56°C for 30s, and extension at 72°C for 40s; and final extension of 72°C for 10 min. Amplicons were run on agarose gel (1.2%), then gel-purified using DNA Gel Extraction kit (Qiagen, Hilden, Germany) following manufacturer's instructions. Amplicons were, then, shipped to Beijing Genome Institute (BGI) in China for library construction and deep sequencing on Illumina Miseq platform. DNA libraries were constructed following the protocol TruSeq DNA sample preparation (Illumina, Inc; San Diego, USA) to recover ∼300 bp pair-end reads of the V3-V4 region. The ends of each read were overlapped to generate high quality, full-length reads. The resulted sequencing data was submitted to European Nucleotide Archive (ENA) (https://www.ebi.ac.uk/ena/submit/sra/#studies) and project number will eventually be given.

### 16S dataset processing:

Sample size estimation was performed to determine the probability that the samples are representative [33]. The raw sequencing data were analyzed using the Quantitative Insights Into Microbial Ecology 2 (QIIME2) package v.2018.11; (https://qiime2.org) [34]. V3-V4 16S rRNA sequence reads were trimmed using trimmomatic software (Version 0.33) and merged into single sequences using FLASH program (Version 1.2.10). Merged sequences were filtered to remove the low-quality sequences. The latter comprise the reads shorter than 100 nucleotides, reads truncated at any site with an average quality score of <20 over a 50-bp sliding window, or the truncated reads that were shorter than 50 bp. Only sequences that overlapped for more than 10 bp were assembled. The unique sequence set was linked to tags and classified into operational taxonomic units (OTUs) with a cutoff of 97% identity using the de novo OTU selection strategy. We retained only OTUs with at least 0.01% mean relative abundance, as predominant. OTUs were ranked by the relative abundance values of x and y-axis, then the rank curve was drawn by software R (Version 3.1.1). Taxonomies were assigned by RDP classifier (Version 2.2) [35,36] and the Greengenes database [37] with a confidence threshold of 0.7. Chimeric sequences were removed using Usearch (Version 8.0).

### Diversity measurements:

Alpha diversity was assessed by Shannon and Simpson indices that were calculated by Mothur (v1.31.2), and the corresponding rarefaction curve was drawn by software R (Version 3.1.1). Drawing rarefaction curve was based on calculating OTU numbers of the extracted tags (in multiples of 500) and detecting the maximum depth (no. reads) permitted to retain all samples in the dataset. Sequences were extracted randomly according to the minimum sequence number for all samples, and the extracted sequences formed a new 'OUT table biom' file. To detect beta diversity within and between groups, weighted and unweighted UniFrac distances were calculated [38] and plotted via principal coordinate analysis (PCoA) using package 'ade4' of software R (Version 3.1.1). UniFrac uses system evolution information to compare composition of community species between samples. Results can be used as a measure of beta diversity. It takes into account the distance of evolution between species, and the bigger the index, the greater the differences between samples. UniFrac is divided into weighted UniFrac and unweighted UniFrac of which the weighted UniFrac considers the abundance of sequences, while unweighted UniFrac gives more weight on species presence/absence. Heat maps were generated using the package 'gplots' of software R (Version 3.1.1). The used distance algorithm is 'euclidean' and the clustering method is 'complete'. At phylum level, all species were used to draw the heat map and taxa of which abundance is less than 0.5% in all samples were classified as 'others'. To minimize the differences degree of relative abundance value, values were all log transformed. The representative sequences were aligned against the Silva core set [39] built-in scripts including fast-tree method for tree construction. The tags with the highest abundance of each genus was chosen as the corresponding genus representative sequences, and genus- level phylogenetic tree was obtained by the same way of OTU phylogenetic tree. Then, the phylogeny tree was imaged by software R (Version 3.1.1). Venn diagram was drawn by software R (Version 3.1.1), while differences in the relative abundances of taxa at the phylum, genus and species levels were analyzed using Metastats [40]. PERMANOVA was used to test significance among values. All statistical tests were two-sided, and P value ≤ 0.05 was considered significant. Benjamini-Hochberg false discovery rate (FDR) correction was used to correct for multiple hypothesis testing where applicable.

## Results

### Statistics of 16S rRNA sequence datasets:

**Figure 1:**
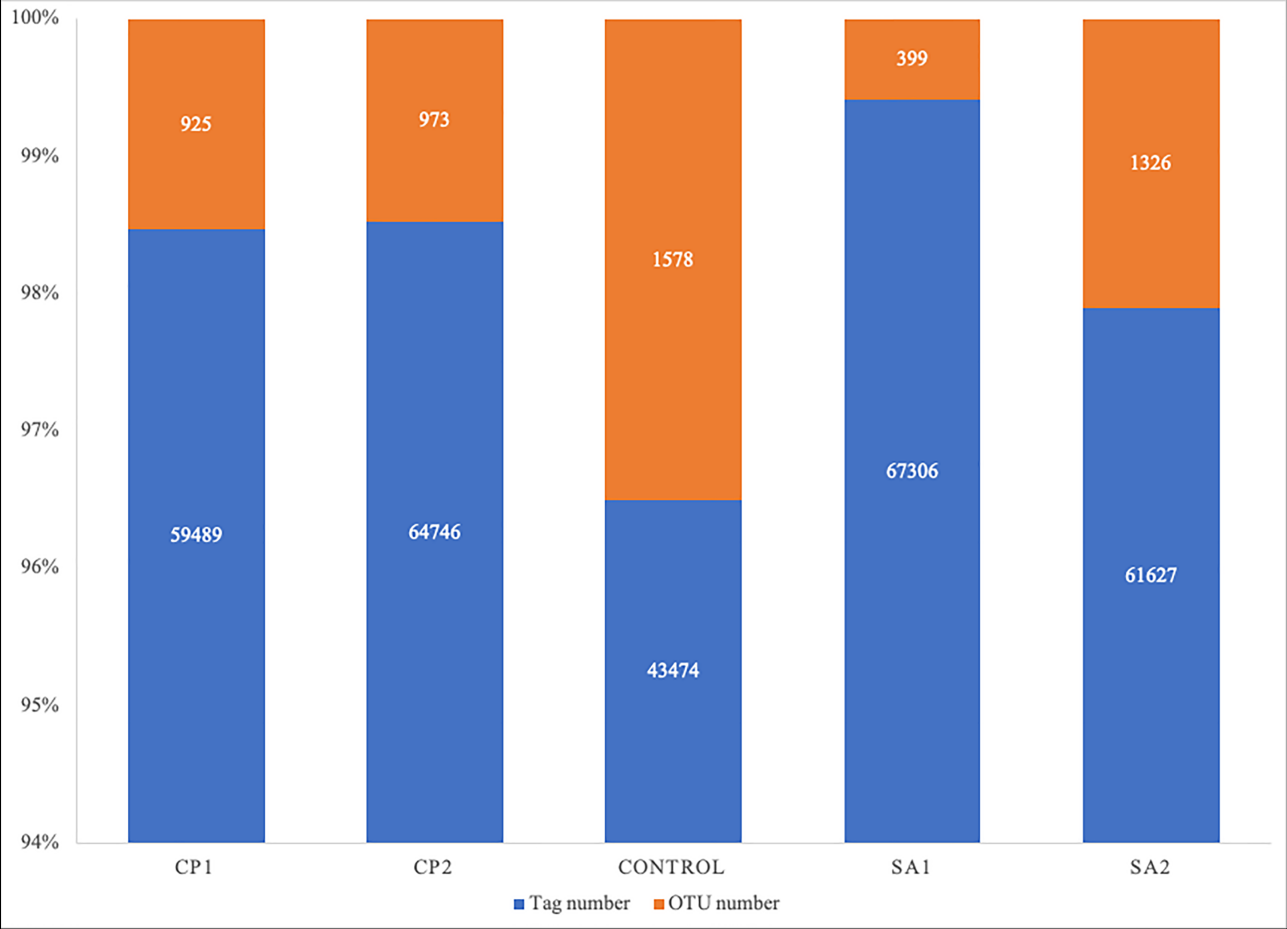
Recovered numbers of tags and OTUs from soil microbiomes collected from the rhizosphere of Calotropis procera (CP) and Senna alexandrina (SA) as well as plant-free microbiome (control).

In the present study, bacterial rhizosphere of two desert plants and one bulk soil were used. Illumina MiSeq was used in analyzing the five samples belonging to three groups based on 16S rRNA. Statistics of the raw data description and its processing is shown in Table 1. The average sequence length per read was 297 bp across different samples ranging from 293 to 300 bp and generating a total of 350,807 clean sequences reads across all samples. A total of 296, 642 tag number were generated across all samples with average read number of 62,117 per CP (Calotropis procera) samples and 64,466 per SA (Senna alexandrina) samples comparing with 43,474 tags per control sample. These sequence tags were assigned to a total of 5,210 OTUs (operational taxonomic units) across samples with ≥ 97% similarity and an average of 949 OTUs per CP and 863 OTUs per SA comparing with 1,578 OUTs per control (Figure 1 and Table 1).

### Diversity of rhizosphere microbiota:

**Figure 2:**
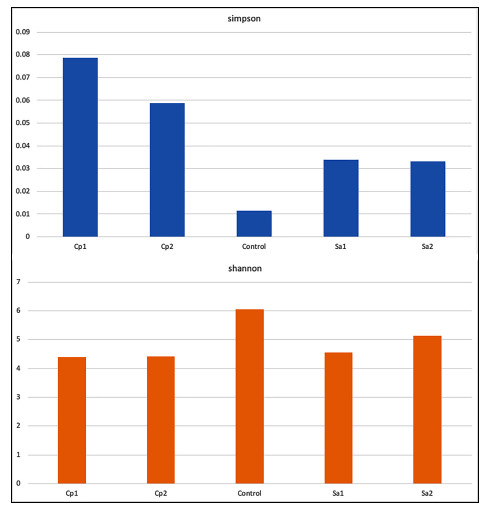
Alpha diversity measures mainly describing richness (Shannon) and evenness (Simpson) in microbes of different soil samples. CP = Calotropis procera, SA= Senna alexandrina.

**Figure 3:**
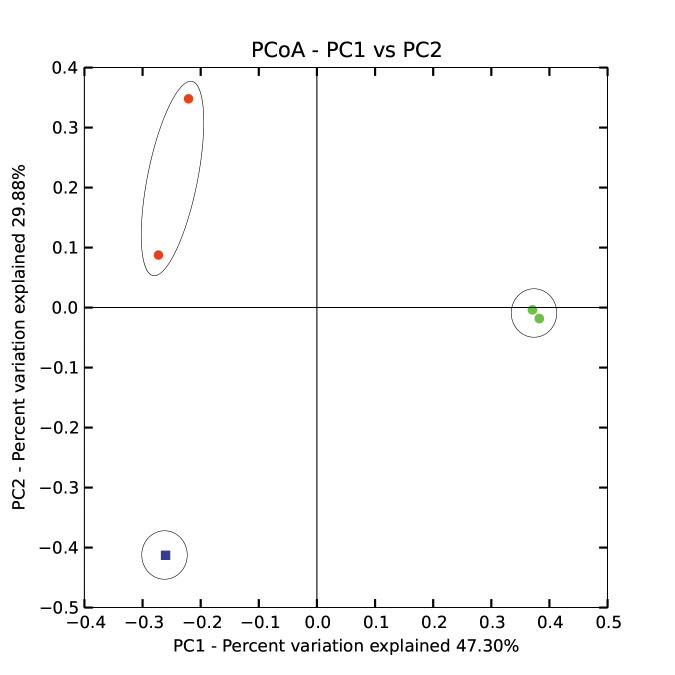
Plot of principal coordinate analysis (PCoA) describing relatedness of microbiomes of the three groups of samples. CP = Calotropis procera, SA= Senna alexandrina.

Alpha-diversity metrics were compared among different soil samples (Figure 2). Shannon index in the rhizosphere of S. alexandrina was higher than that of C. procera, while lower than in the corresponding bulk soil. Simpson index indicated opposite results. This indicates the high richness in control sample than those collected from rhizosphere of either plant. This conclusion aligns with the assumption that plants reduce richness of microbes by allowing growth of selective microbes most likely beneficial. However, other environmental parameters (such as soil texture, pH, etc.) might also contribute to the growth rates of microbes. Principal coordinate analysis (PCoA) was done to describe differences within and among the three groups (Figure 3). PCoA plot indicated complete separation of microbiome diversity among groups. Diversity of the two C. procera samples was located towards positive direction of PCoA 2 direction (PC2), while negative direction of PC1. Diversity of the two S. alexandrina was located in the positive direction of PC1 with no tendency towards a certain position in the PC2 direction. Bulk soil (control) was separated towards negative directions of both PC1 and PC2.

**Figure 4:**
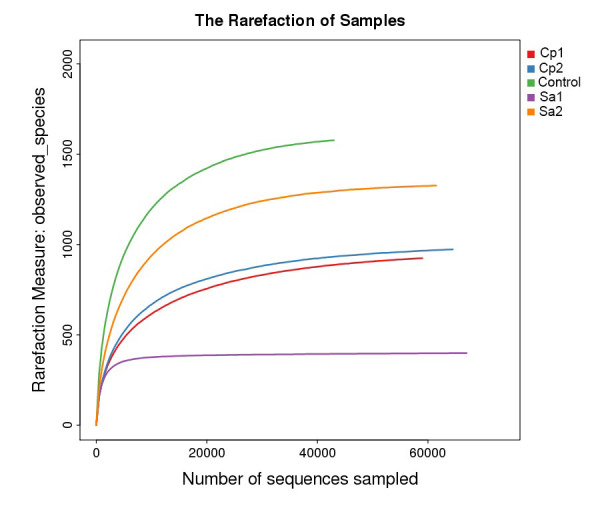
Rarefaction curves describing number of OTU tags used as cutoff for subsequent beta diversity analysis. Arrow refers to the maximum depth permitted to retain all samples in the dataset. CP = Calotropis procera, SA = Senna alexandrina.

Rarefaction curves across the five-microbiome samples based on number of OTU tags were drawn (Figure 4). Cutoff used as rarefaction measure describing the maximum depth permitted to retain all samples in the dataset for studying taxonomic relative abundance was 54,000 sequence tags. The more the curve continues to climb with increasing sequencing reads, the higher the complexity in samples that better describe diversity of samples.

### Taxonomic Composition of the highly abundant microbes:

**Figure 5:**
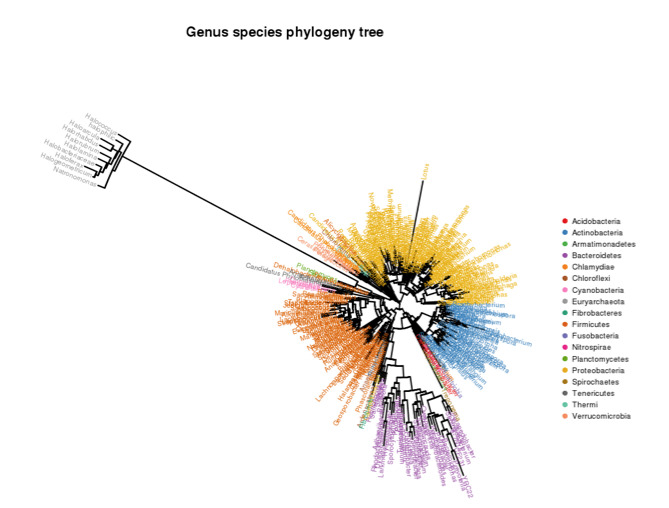
Phylogenetic tree describing genera and species in microbiomes across the three groups of samples, e.g., rhizospheres of Calotropis procera and Senna alexandrina as well as soil bulk control. Original data is shown in Table S1.

Taxonomic composition of rhizosphere microbiomes of the two wild plants along with their control soil microbiome is shown in Table S1. Overall, 3,420 prokaryotic OTUs were identified in the rhizosphere samples and bulk soil. Phylogenetic tree describing microbiome taxonomic groups of rhizosphere and bulk soil at the genus levels is shown in Figure 5. A phylogenetic tree is a branching diagram showing the inferred evolutionary relationships among various biological taxa based upon similarities and differences in their physical or genetic characteristics. The evolution distance between taxa is closer if the branch length is shorter. The results indicated that the most common phyla are Proteobacteria (128 genera), Firmicutes (81 genera), Actinobacteria (53 genera), Bacteroidetes (42 genera), Verrucomicrobia (six genera), Chlamydiae (four genera), Chloroflexi (three genera) and Tenericutes (three genera) (Figure 5 and Table S1).

**Figure 6:**
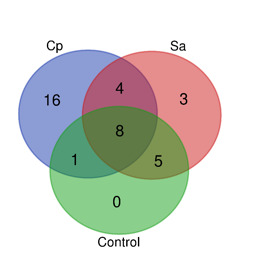
Venn diagram describing highly enriched unique and shared microbes of the three groups of samples. The diagram refers to highly enriched soil microbes or OTUs (37) with cut-off of ≥ 1000 reads. CP=Calotropis procera, SA=Senna alexandrina. Original data is shown in Table S1. CP was observed to share (1+8) 9 OTU's with control, 8 of which were also shared with SA, but CP and Sa shared a total of (8+4) 12 OTU's. SA in turn shared (8+5) 13 OTUs with control. Of the total 37 OTU's recorded, 8 were shared by CP, SA and control. Numbers of 16 and 3 microbes were uniquely found in microbiomes of CP and SA, respectively.

**Figure 7:**
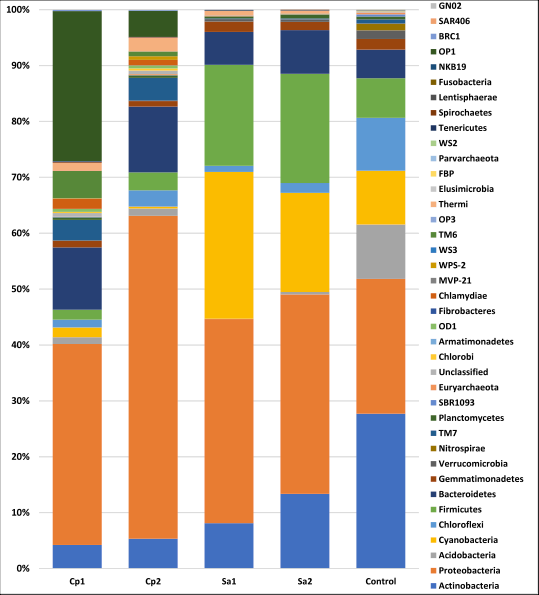
Differential abundance of microbes among samples at phylum level. CP = Calotropis procera, SA= Senna alexandrina.

**Figure 8:**
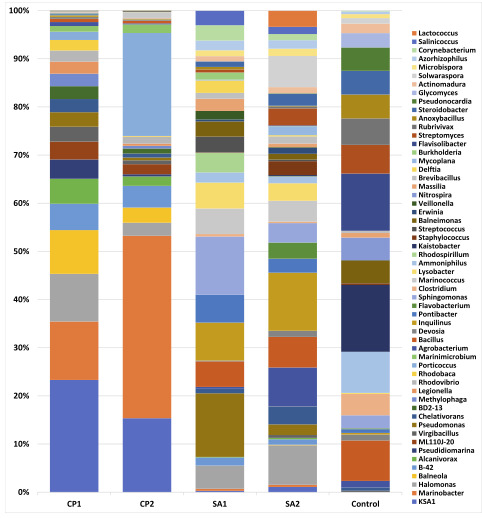
Differential abundance of microbes among samples at genus level. CP = Calotropis procera, SA= Senna alexandrina.

In terms of highly abundant microbes, results shown in Figure S4 displaying beta diversity heat maps of weighted and unweighted unifrac diversity distances among the three groups indicated a complete separation of Calotropis procera samples and partial relationship between Senna alexandrina and control plant-free sample. Assignment of highly abundant bacterial OTUs revealed the presence of 39 phyla (Figure 6 and Figure S5), 56 genera (Figure 7 and Figure S6) and nine species (Figure 8 and Figure S7). At phylum level, Calotropis procera rhizosphere differed in bacterial community composition from Senna alexandrina rhizosphere and bulk soil, where Proteobacteria and Bacteroidetes increased in one of the two rhizosphere samples of C. prociera compared with those of S. alexandrina or plant-free control. Opposite results were reached for Cyanobacteria, Actinobacteria and Firmicutes that enriched in the rhizosphere of S. alexandrina. Abundance of microbes of Bulk soil sample stood in the middle between the two rhizospheres at phylum level (Figure 6 and Figure S5). In terms of shared microbes at genus level, none increased in rhizosphere of C. procera although Sphingomonas genus increased in that of S. alexandrina. Kaistobacter genus was highest in control plant-free sample (Figure 7 and Figure S6). At species level, Pseudomonas stutzeri and Virgibacillus koreensis increased in the rhizosphere of C. procera compared with that of S. alexandrina. Opposite results were reached for Streptococcus sobrinus and Veillonella parvula. Anoxybacillus kestanbolensis and Actinomadura vinacea were highest in control plant-free sample (Figure 8 and Figure S7). Interestingly, unassigned species of the genus Pseudomonas was highest in rhizosphere of S.alexandrina, while Pseudomonas stutzeri was highest in rhizosphere of C. procera.

**Figure 9:**
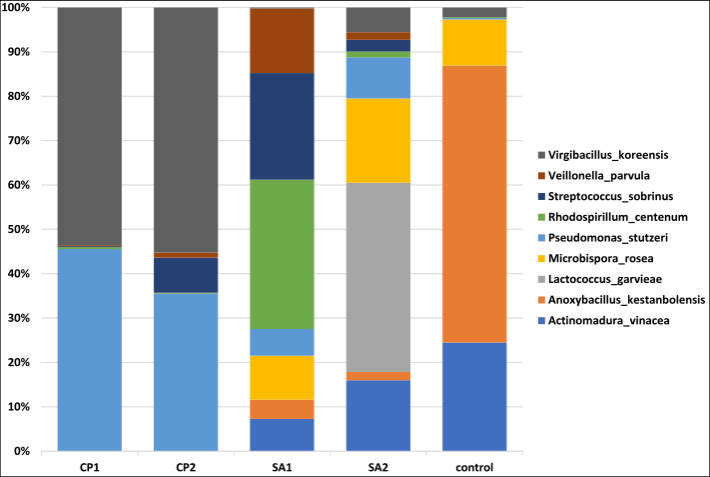
Differential abundance of microbes among samples at species level. CP=Calotropis procera, SA=Senna alexandrina.

Highly enriched soil microbes or OTUs [37] with cutoff of ≥ 1000 reads were further analyzed (Table S1). Venn diagram describing occurrence of highly enriched unique and shared microbes is shown in Figure 9. The diagram indicated exclusive presence of bacterial taxa in rhizosphere of one or the two wild plants comparing with control plant-free sample, where microbiome rhizosphere of Calotropis procera resulted in the occurrence of 16 taxa, while that of Senna alexandrina resulted in the occurrence of three microbes (Figure 9 and Table S1). No microbes exclusively present in plant-free control microbes. None of highly enriched microbes was assigned at species level. At genus level, unassigned species of genera Marinobacter, Porticoccus and Alcanivorax only exist in rhizosphere microbiome of Calotropis procera, while unassigned species of genus Pseudomonas only exists in rhizosphere microbiome of Senna alexandrina. In addition, genus Halomonas exists in rhizosphere microbiomes of the two wild plants (Table S1). In summary, abundances of Pseudomonas stutzeri and Virgibacillus koreensis increased in the rhizosphere of C. procera compared with that of S. alexandrina, while abundances of Streptococcus sobrinus, Veillonella parvula and unassigned species of Sphingomonas genus increased in rhizosphere of S. alexandrina. Anoxybacillus kestanbolensis, Actinomadura vinacea and unassigned species of Kaistobacter genus were highest in control plant-free sample. In terms of exclusive presence of microbes in one group, unassigned species of genera Marinobacter, Porticoccus and Alcanivorax only exist in rhizosphere microbiome of C. procera, while unassigned species of genus Pseudomonas only exists in rhizosphere microbiome of Senna alexandrina. In addition, genus Halomonas exists in rhizosphere microbiomes of the two wild plants.

## Discussion

Interest in studying microbial diversity of desert plants rhizosphere is increasing [41,42] as this habitat is severely influenced by global climate changes in which arid regions like those in the KSA is more vulnerable. Deep sequencing of V3-V4 region of 16S rDNA gene from the rhizospheric plant samples (e.g., C. procera and S. alexandrina) and bulk soil sample (control) from the desert of Makkah region (Saudi Arabia) revealed a large bacterial biodiversity in these harsh conditions. We obtained 350,807 high quality sequences, which are classified from the phylum to species levels. It is important to note that the five samples came from the same site and included the rhizospheres of two different pioneer plants in this region. Samples showed high level of unassigned species of a large number of genera. This reflects the native nature of the selected location of the study. We expect that culturing of these new microbes will be moderately successful.

The variety of organic compounds released by plants is postulated to be a main factors affecting the diversity of microorganisms in the rhizosphere of these plants [41, 43]. We examined the bacterial richness and diversity in each sample using Shannon and Simpson estimators and found a large inter-sample variability within (319 to 961 OTUs for the two replicates of S. alexandrina) or across rhizospheres and control samples. These results suggest that the number of sequences generated from high throughput sequencing is not always a limiting factor for estimating the total bacterial diversity.

Although richness in plant-free sample was higher than those of the four samples of rhizosphere microbiomes (Table 1), no exclusive growth of microbes in the plant-free soil was detected referring to the highly enriched microbes. This indicates that interaction between plant roots and microbes is a selective process and plant exudates seem to allow growth of some microbes and block growth of others. Plant-free condition does not encourage bacteria to grow well, which indicates the necessity for the symbiotic relationship between microbes and plant for ideal microbial growth on one hand, and possibly better plant growth on the other hand.

In terms of microbes with differential abundance, Pseudomonas stutzeri and Virgibacillus koreensis increased in the rhizosphere of C. procera. Pseudomonas stutzeri was proven to promote plant growth under saline stress [44]. One strain of this species showed extremely positive chemotaxis towards root exudates and the ability to form biofilm on soybean roots under high saline conditions. The microbe has a positive influence on seed germination, plant growth and general plant health. Earlier studies indicated that this microbe has important properties such as degradation of aromatic compounds, denitrification, and nitrogen fixation [45]. Virgibacillus koreensis was originally isolated from a salt field [46]. Virgibacillus species are generally halophillic and possess the ability to solubilize phosphate and produce auxin, important characteristics of plant growth promoting rhizobacteria (PGPR) [47]. We concluded that high abundances of the two microbes Pseudomonas stutzeri and Virgibacillus koreensis in rhizosphere of C. procera allow the plant to grow well under both normal and saline condition.

As indicated earlier, abundances of Streptococcus sobrinus, Veillonella parvula and unassigned species of Sphingomonas genus increased in rhizosphere of S. alexandrina. Interestingly, Streptococcus sobrinus [48] and Veillonella parvula [49] were reported as pathogens to human and induce biofilm formation in patients with dental caries [48] and [49] respectively. We have no explanation for the presence of these two microbes in the rhizosphere of S. alexandrina. On the other hands, Sphingomonas genus generally promotes the growth of Arabidopsis by driving developmental plasticity in the roots and stimulating growth of lateral roots and root hairs besides its ability to degrade organic pollutants [50]. The latter microbe justifies the plant’s ability to grow well and stand water scarcity.

Three microbes existed in plant-free sample. They include Anoxybacillus kestanbolensis and Actinomadura vinacea and unassigned species of Kaistobacter genus. A. kestanbolensis is a thermophilic bacillus originally isolated from mud and hot springs with ability to grow on a wide range of carbon sources [51] and was also proven to possess important thermo- and alkalostable catecholases [52]. The second microbe is an animal pathogen that was isolated from a nonhealing cutaneous wound of a cat [53]. These two microbes were not found in rhizospheres of any plant up to date aligning with the data of the present study. However, unassigned species of Kaistobacter genus is among microbes recently used as synthetic fertilizer [54]. So, this microbe is expected to grow better around plant roots. Further studies might be required to detect the exact host-microbe relationships referring to this genus.

In terms of highly abundant microbes with cutoff of ≥ 1000 reads, results indicated that unassigned species of Marinobacter, Porticoccus and Alcanivorax genera only exist in rhizosphere microbiome of Calotropis procera. Marinobacter is a member of the gamma group of the Proteobacteria. The three genera act on degrading hydrocarbons. Marinobacter hydrocarbonoclasticus was reported to produce the petroleum-biodegrading siderophore petrobactin [55,56]. This microbe can form biofilms on hydrophobic organic compounds and degrade hydrocarbons, which make this species a research interest in the field of marine ecology [57,58]. In general, microbial siderophores have a major role in remediation of petroleum hydrocarbons from marine environments [59]. Rhizosphere siderophores protect plant from pathogens by blocking availability of iron ions to pathogenic organisms. Porticoccus hydrocarbonoclasticus is also able to degrade three-and four-ring polycyclic aromatic hydrocarbons PAHs [60], while Alcanivorax is the first bacteria to flourish on a wide range of alkanes after an oil-spill [61]. This genus blooms right after superficial oil spills, reaching about 80-90% of the total bacterial community [62]. On the other hand, unassigned species of Pseudomonas existing in rhizosphere microbiome of Senna alexandrina has a negative influence on plant immune system as it can suppress local plant defense and trigger expression of microbe-associated molecular patterns (MAMP)-inducible genes [63]. The results for the exclusive presence of microbes around the two plant species indicate that C. procera might be more protected from microbial pathogens compared with S. alexandrina due to the microbes growing in rhizospheric region.

The genus Halomonas exist in rhizosphere microbiomes of the two wild plants, but not in plant-free control (Figure 8 and S6). This genus is extremely salt-tolerant and participates with other microbes in forming biofilms that is associated with soil adherence to plant roots [64, 65]. Interrelationships with plant roots in terms of function were not proven to affect salt stress tolerance in plants. Further analysis might be required to illustrate functions acquired by plant due to presence of these microbes in their rhizosphere. Moreover, more replicates are recommended in future work to detect microbial abundances at statistical level.

## Conclusion

C. procera is shown protected from microbial pathogens and more tolerant to abiotic stresses compared with S. alexandrina due to the microbes growing in rhizospheric region. These results indicate that rhizospheric microbes can be considered as biomarkers of plant growth rate as well as its ability to survive under harsh conditions.

## Declaration on Publication Ethics:

The authors state that they adhere with COPE guidelines on publishing ethics as described elsewhere at https://publicationethics.org/. The authors also undertake that they are not associated with any other third party (governmental or non-governmental agencies) linking with any form of unethical issues connecting to this publication. The authors also declare that they are not withholding any information that is misleading to the publisher in regard to this article.

The authors are responsible for the content of this article. The Editorial and the publisher has taken reasonable steps to check the content of the article with reference to publishing ethics with adequate peer reviews deposited at PUBLONS.

## Figures and Tables

**Table 1:** The data generated from deep sequencing for soil microbiomes collected from the rhizosphere of Calotropis procera (CP) and Senna alexandrina (SA) as well as plant-free microbiome (control).

Sample name	Reads length (bp)	Raw reads	Clean reads	% Read utilization	Tag number	OTU number
CP1	297:296	73196	69996	95.63	59489	925
CP2	299:296	73382	70239	95.72	64746	973
SA1	298:296	73965	70958	95.93	67306	399
SA2	300:296	72935	70101	96.11	61627	1326
Control	300:300	72167	69513	96.32	43474	1578
